# Alcoholism and Intimate Partner Violence: Effects on Children’s Psychosocial Adjustment

**DOI:** 10.3390/ijerph6123156

**Published:** 2009-12-10

**Authors:** Keith Klostermann, Michelle L. Kelley

**Affiliations:** 1 School of Nursing, University of Rochester, Box SON, 601 Elmwood Avenue, Rochester, NY 14647, USA; 2 Old Dominion University, 134C Mills Goodwin Building, Norfolk, VA 23529-267, USA; E-Mail: mkelley@odu.edu

**Keywords:** domestic violence, partner aggression, children’s adjustment

## Abstract

It is widely recognized that alcoholism and relationship violence often have serious consequences for adults; however, children living with alcoholic parents are susceptible to the deleterious familial environments these caregivers frequently create. Given the prevalence of IPV among patients entering substance abuse treatment, coupled with the negative familial consequences associated with these types of behavior, this review explores what have been, to this point, two divergent lines of research: (a) the effects of parental alcoholism on children, and (b) the effects of children’s exposure to intimate partner violence. In this article, the interrelationship between alcoholism and IPV is examined, with an emphasis on the developmental impact of these behaviors (individually and together) on children living in the home and offers recommendations for future research directions.

## 1. Introduction

Although historically viewed as a private family matter, for more than 30 years, intimate partner violence (IPV) has been recognized as a broad societal problem that necessitates the attention of both the mental health and criminal justice systems. According to data gathered as part of the National Crime Victimization Survey, in 2005, there were approximately 3.5 million reports of family violence and nearly 1 million female victims of intimate partner violence [1]. Moreover, estimates indicate that approximately 15–20% of partners engage in IPV at least once annually [2]. In addition, the National Center for Victims of Crime reports 32% of college students have experienced violence in a previous relationship and 21% report violence in their current relationship [3]. Acts of IPV range from beating up one’s partner repeatedly to a single episode of pushing one’s partner [2,4–6].

IPV often has serious public health consequences. According to the Department of Justice (DOJ) estimates, over 400 men and 1,200 women are killed by an intimate partner each year [1]. In fact, partner violence accounts for 11% of all homicides annually [6]. In addition, IPV often results in acute injuries (e.g., bruises, lacerations, broken bones and teeth) and may result in chronic illnesses and psychiatric conditions (e.g., chronic pain, substance use; [7,8]). Specifically, a 2001 Department of Justice (DOJ) report indicated there were 691,000 nonfatal violent victimizations committed by current or former spouses, boyfriends, or girlfriends each year. Moreover, nearly a quarter million emergency room visits each year involve a victim of IPV. According to the findings of the National Crime Victimization Survey [1], between 2001 and 2005, nonfatal intimate partner victimizations accounted for 22% of victimizations against women age 12 and older. In addition to the physical consequences, victims of IPV also may experience depression, substance abuse, anxiety, and low self-esteem as a result of the victimization. Related to this, in the United States alone, the economic costs associated with IPV against women is estimated to range from $2.3 billion to $7 billion per year [9].

Results of prior investigations, in a variety of settings, provide evidence of a link between the occurrence of IPV episodes and substance use (*i.e*., comorbidity model). Although both substance use and partner violence are viewed as observable manifestations of a common set of problems, neither is believed to be a cause of the other [10]. In a national sample of 5,159 families, Kaufman Kantor and Strauss [11] Kaufman Kantor and Strauss found over 20% of males and 10% of females were drinking prior to the most recent and severe act of violence. In the National Crime Victimization Survey [12], 43% of the victims of IPV reported the perpetrator had been under the influence of drugs. Studies of college student populations, which have often focused on forms of IPV that involve sexual violence, have found that 50% of assaults involve alcohol use [13]. Among prisoners convicted of murdering an intimate partner, 45% reported that they were drinking at the time of the incident, with an average blood alcohol concentration of 3 times the legal limit. For married or cohabiting patients entering treatment for alcoholism and other drugs of abuse, the proportion of these dyads reporting at least one episode of IPV in the previous year is 4–6 times higher than observed in national samples [14,15]. In addition, the strong relationship between alcohol use and perpetration of IPV has been found in primary health care settings [16], family practice clinics [17], prenatal clinics [18], and rural health clinics [19]. Yet, as noted by Gil-Gonzalez *et al.* [20] in their meta-analysis of studies examining the alcohol and partner violence link, these findings must be interpreted with caution since many of these study designs lack inferential power and there is also a possibility of publication bias. As consistent and powerful as these findings may be, more rigorous study is needed on this phenomenon.

It is widely recognized that alcoholism and relationship violence often have serious consequences for adults (e.g., emotional, economic, behavioral, physical, social); however, children living with alcoholic or drug-abusing parents are susceptible to the deleterious familial environments these caregivers frequently create. Although many genetic and environmental factors may increase offspring risk, and this is not to say that other factors (*i.e.*, parenting practices, peer values, neighborhood influences) should not be examined in tandem, IPV in the context of parental alcohol abuse plays a very significant, but often overlooked, role in children’s short- and long-term outcomes. Thus, our argument, theoretical explanations, and suggestions for future research focus on the need for researchers to establish how children’s exposure to IPV and parental alcohol misuse may individually or in combination create risk for negative psychosocial outcomes. Moreover, given the clear evidence for the relationship between parental alcohol abuse and IPV, for perhaps the majority of children, mental health treatment should address both the potential harm that exposure to interparental violence and parental alcoholism create.

Given the prevalence of IPV among patients entering substance abuse treatment, coupled with the negative familial consequences associated with these types of behavior, this review will further explore what have been, to this point, two divergent lines of research: (a) the effects of parental alcoholism on children, and (b) the effects of children’s exposure to intimate partner violence. Separate literatures have evolved because in general the majority of studies have compared children of alcoholics (COAs) to non-COAs and children who witness interparental violence to controls. As such, first, we explore the impact of parental alcoholism on children’s development. Second, we examine the effect of IPV on children living in these environments. Third, we highlight theories that may help to explain the effects of IPV, parental alcoholism, and the interaction of these variables on child outcomes. Finally, we provide a brief argument for investigating IPV in the context of alcohol use disorder, which may be especially detrimental for children’s mental health treatment.

## 2. Parental Alcohol Abuse and Child Development

Although alcoholism often has serious emotional, economic, behavioral, physical, and social consequences for alcohol abusers and their partners, children who live with alcoholic parents often experience negative psychosocial outcomes. In general, the literature supports that COAs are more likely to develop externalizing problems such as conduct disorder, oppositional defiant disorder, delinquency, and attention deficit disorder (e.g., [21,22]), and are at elevated risk for internalizing behaviors such as depression and anxiety [23,25]. In addition, offspring of alcoholics drink earlier (e.g., [24]), are more likely to develop alcohol use problems [25–28], progress from initial alcohol use to alcohol use disorder more quickly [25,28], and are less likely to mature out of moderate to heavy drinking [30].

Although, in general, the literature supports an elevated risk for negative psychosocial development among children with a family history of alcoholism (e.g., [31]), many children who live with an alcohol-abusing parent display normal psychosocial development (e.g., [32–34]). With respect to parental alcohol abuse and IPV, Nicholas and Rasmussen [33] found that when they controlled for childhood abuse and IPV, parental alcohol use did not predict reports of aggression or depression among college-student adult children of alcoholics (ACOAs).

Undoubtedly, characteristics of the family environment contribute to the adjustment of COAs. Thus, the challenge for researchers is to refine the definition of risk by identifying specific mechanisms that lead to diverse outcomes among children raised by alcoholic parents. Although alcohol misuse during gestation has well-documented risk for physical and central nervous system insults that may result in cognitive, affective, growth, and morphologic sequelae [34], our theoretical explanations focus on factors relevant to alcohol-abusing couples that with intervention may be most amenable to change.

### 2.1. The Effects of IPV on Children in Their Homes

The Department of Justice estimates that 3.3 to 10 million children are exposed to domestic violence annually [1]. McDonald *et al.* [36] found that approximately 15.5 million children live in households where IPV has occurred, with seven million living in homes where severe forms of partner aggression has occurred [36]. Although differences in the measurement of IPV have resulted in variability in the estimates of children’s exposure to IPV, regardless of the definition of IPV, these figures may underestimate the true magnitude of the problem.

Witnessing severe interparental conflict has been linked to children’s feelings of terror and helplessness, fears for their own and their parents’ safety [37], and children’s depression, anxiety, somatic complaints, and sleep disruptions [38–41]. Cummings and Davies [42] contend that children evaluate marital conflict in terms of its implications for their emotional security and respond accordingly. Thus, IPV may affect children’s emotional security, and, in turn, may increase youth risk for adjustment problems [43]. In addition, chronic exposure to parental violence may also result in structural changes to a child’s frontal temporal lobe, resulting in difficulty organizing thoughts and problem-solving [44]. These changes in the brain chemistry may manifest themselves in terms of hypervigilant behaviors (e.g., keenly aware of gestures and sounds as possible violence indicators).

In addition to the harmful effects that IPV may have on children’s emotional adjustment, exposure to IPV, and the victimization that children experience from their exposure to IPV, increase children’s proneness to bullying, aggressive, violent, and delinquent behavior [40,45–49]. Flannery, Singer, and Wester [50] found dangerously violent girls were 2–7 times more likely to have been exposed to violence, and were 3–5 times more likely than controls to have scored in the clinical range of depression, anxiety, posttraumatic stress, anger, and dissociation. Dangerously violent boys were 3–6 times more likely than controls to have been a victim of, or witness to violence [50]. Moreover, adolescent boys exposed to IPV are more likely to believe that use of aggression is acceptable in romantic relationships [51], and engage in more aggressive behaviors with their romantic partners [51,52]). It is important to recognize that these negative behavioral and developmental outcomes are independent of any direct abuse or neglect they may have also experienced from parental figures [53].

### 2.2. IPV, Parental Alcohol Abuse, and the Development of Maladaptive Behavior in Children: Theoretical Perspectives

Alcoholism and IPV often occur together; however the relationship between the two issues is complex and not well understood [54]. As a result, a number of theories have been proposed to explain how parental alcohol abuse may create risk for custodial children in their homes. For example, Social learning [55,56] and theories of Social development [57,58] stress the importance of socialization and healthy relationships with parents and others to model prosocial beliefs and behaviors, and to provide interactions that illustrate appropriate rewards and consequences. Developmental ecological approaches have been used to conceptualize risk for antisocial behavior [59]. According to family-couple theories, interparental conflict is the primary mediating pathway leading to child adjustment problems. Finally, Spillover theories contend that interparental conflict is linked to family processes and parenting [60–62]. Given the heterogeneity in the family environments, neighborhoods, and temperament of COAs, many theories may help explain youth development.

It is important to note that these diverse conceptualizations of mechanisms of action underlying child risk are not mutually exclusive; simply stated, each of these theories includes elements of one or more of the others in its overall model of the manner in which children’s emotional and behavioral problems evolve. However, differences exist in the way each paradigm explains the various destructive factors that may operate to influence negative child outcomes. For example, while there is much agreement that parental alcoholism and intimate partner violence are serious destructive influences in children’s development across the various theories, the role of each is different depending on the manner in which each of these behaviors are conceptualized.

### 2.3. Social Learning and Social Development Theories

According to social learning theory, problematic drinking and violent behavior are learned primarily through social interactions, which are passed down from one generation to the next. In particular, exposure to violence between parents may teach children that violence is an acceptable means of conflict resolution [63]. Thus, an individual may have acquired (learned) poor coping strategies (*i.e.*, drinking and violence) through modeling dysfunctional behavior exhibited in the family of origin. Social learning theories may be helpful in explaining patterns of intergenerational violence.

### 2.4. Developmental Ecological Approaches

A developmental ecological framework would argue that the contexts created by parental alcohol use may expose COAs to greater developmental risk. For instance, both legal problems [64,65] and unemployment [65,66] are related to adult alcohol abuse. Moreover, alcohol abuse may jeopardize marital relationships [67] and increase negative affect [68].

It is now widely accepted that the occurrence of violence between intimate partners is the culmination of multiple interacting contextual, social, biological, psychological, and personality factors that exert their influence at different times, under different circumstances, acting in a probabilistic fashion [69]. Consequently, ecological models examine these factors on multiple levels. From an ecological vantage, there are four levels of analysis: (1) personal history factors the individual brings into the relationship, (2) the immediate context in which the abuse takes place (*i.e.*, microsystem; e.g., family, intimate partner), (3) institutions and social structures that comprise the microsystem (e.g., work, neighborhood, social networks), and 4) macrosystem; the general views and attitudes that permeate the culture at large [70]. In addition to these four areas, Edleson and Tolman [71] also consider a fifth factor, the mesosystem, which includes the interrelationship among the various factors identified above (e.g., link between person’s family and employment, relationships with legal institutions, social groups).

Ecological theories have been used to explain how youth who experience parental alcohol abuse and IPV may be more likely to live in high-crime neighborhoods which may adversely impact the quality of schools and increase exposure to neighborhood violence. These parents may not be able to protect their children from neighborhood influences by moving to a safer area. The developmental ecological approach emphasizes both the social ecology in which the child develops, particularly for youth and families in high-risk settings (e.g., [72]), and risk factors that vary depending on child age [73].

### 2.5. Family-Couple Theories

Viewed from a family-couple vantage, witnessing paternal alcoholism and intimate partner violence has been linked to children’s fears [37] and internalizing symptoms [38–41]. The combined verbal/physical dyadic violence has also been related to greater likelihood of aggression and emotional maladjustment in children [74]. Moreover, growing empirical evidence shows that childhood exposure to the trauma of others can compromise adolescent and adult mental health outcomes [75]. Because dyads in which married or cohabiting patients entering treatment for alcoholism and other drugs of abuse are 4–6 times more likely to engage in acts of intimate partner violence than couples in the general population, children in these homes may be exposed to comparatively high levels of partner violence [14,15].

### 2.6. Spillover Theories

In recent years, researchers have recognized that interparental conflict is intrinsically and empirically linked to family processes and parenting [60–62]. Although there are different forms of interparental conflict [62], the overt hostile style [76], which involves frictional conflict in which couples display verbal aggression and physical violence [77], depicts many couples in which a partner abuses alcohol or drugs [78].

In these couples, poor communication is hypothesized as the mode by which partners communicate and work through everyday disagreements that ‘spill over’ into parent-child interactions and parenting behaviors [79]. In a meta-analytic review of the association between marital quality and parenting, Krishnakumar and Buehler [62] found an average effect size of d = −0.62 between overt interparental conflict and negative parenting. It is possible that parents who engage in intimate partner violence may exhibit an overall style of interaction toward their children that is characterized by coerciveness and negative verbalizations [80].

Each of the theories outlined above may provide a conceptual framework from which to test the effects of IPV and parental alcoholism on youth development. It is important to recognize that a single model may not account for all aspects of child risk.

## 3. Future Directions and Recommendations

Results from epidemiological surveys indicate a significant proportion of school-aged children live in homes in which one or both parents abuse alcohol. More importantly, in addition to the damage caused by parental alcoholism, it appears that these home environments are often marked by high levels of violence and general interparental conflict. Given the prevalence of partner violence among married or cohabiting alcoholic patients, coupled with the number of children living in these homes, future investigations are needed to examine not only the link between alcoholism and partner violence, but also the individual and collective impact these behaviors have on these children.

Unfortunately, alcoholism treatment providers and programs have not raised IPV and its impact on children’s adjustment as an issue, in part because it has gone undetected. Given that the majority of custodial parents who enter treatment for alcoholism are reluctant to allow their children to be involved in any type of mental health treatment, regardless of whether it is individual treatment or as part of family therapy [81], the psychosocial adjustment of a significant cohort of children who live in these homes has been largely ignored. The conspicuous lack of systematic investigations examining the independent and combined relations among parental alcoholism, violence exposure, and children’s adjustment has led Nicholas and Rasmussen [33] to question the legitimacy of continuing to research externalizing behaviors in relation to being a COA if histories of interparental violence are not controlled for in the design. The results of a recent study show that families with documented incidents of domestic violence tend to have multiple young children in the home [38]; coupled with the fact that IPV rates are highest early in the marital relationship (when children are likely to be young; [82]), there is clearly a need to elucidate the factors that influence children’s adjustment. Along with exploring the effects of parents’ alcohol use and IPV on children, examining families with children in which caregivers abuse alcohol and in which children were not exposed in utero may also present an opportunity to examine the effects of postnatal social exposure on children. Furthermore, there is currently a lack of research on the relative risk to children of male- versus female-initiated partner violence; at this time, very little is known about the differential effect [36]. In addition, investigations are also needed to examine the differential impact of violence and alcoholism on male versus female children’s adjustment. Finally, given the heterogeneity in subtypes of violence, future investigations should examine the impact of various types of violence exposure (e.g., severe, nonsevere) on children’s development.

While addressing issues as complex and sensitive as the individual and combined effects of alcohol and IPV (not only between the partners, but also their children), appears overwhelming, given the seriousness and harmful short- and long-term effects of these behaviors, it is critical that the research community begin to examine these issues. For example, important questions such as “What are the interactive effects of these phenomena on children’s adjustment?” and “What is the impact of a reduction in IPV, but not in alcohol use (and *vice versa*)?” have yet to be explored. Further research is also needed to examine the specific mechanisms and how intervention programs might serve to mitigate harm among children from homes with an alcohol-abusing parent and where IPV is present. The results of these investigations will have important implications for the development of treatments necessary to address these complex issues.

## 4. Conclusions

In closing, we believe that concerted efforts are needed to investigate the psychosocial adjustment of children living in violent alcoholic homes. Without a better understanding of the psychosocial adjustment of these children and the factors (e.g., individual, dyadic, parental, biologic, and familial) that may contribute to these home environments, our ability to develop and evaluate treatments with these high-risk families will be greatly impaired. Whether parental alcohol use, coupled with interparental violence may provide unique, interactive, or cumulative risk for children in these homes is not well-understood. While these behaviors are unlikely to be the only risks children in these homes encounter, we strongly believe that each of these behaviors may result in both short-term and potentially longer lasting effects on their development. Ultimately, the knowledge gleaned from these types of investigations will lead to the greatest level of safety for patients, their partners, and their children and aid in developing better policies and treatments [82].
